# Regional disaster risk management strategies for food security: Probing Southern African Development Community channels for influencing national policy

**DOI:** 10.4102/jamba.v10i1.468

**Published:** 2018-05-03

**Authors:** Happy M. Tirivangasi

**Affiliations:** 1 Department of Sociology and Anthropology, University of Limpopo, South Africa

## Abstract

Natural disasters and food insecurity are directly interconnected. Climate change related hazards such as floods, hurricanes, tsunamis, droughts and other risks can weaken food security and severely impact agricultural activities. Consequently, this has an impact on market access, trade, food supply, reduced income, increased food prices, decreased farm income and employment. Natural disasters create poverty, which in turn increases the prevalence of food insecurity and malnutrition. It is clear that disasters put food security at risk. The poorest people in the community are affected by food insecurity and disasters; hence, there is a need to be prepared as well as be in a position to manage disasters. Without serious efforts to address them, the risks of disasters will become an increasingly serious obstacle to sustainable development and the achievement of sustainable development goals, particularly goal number 2 ‘end hunger, achieve food security and improved nutrition and promote sustainable agriculture’. In recent years, countries in southern Africa have experienced an increase in the frequency, magnitude and impact of climate change–related hazards such as droughts, veld fire, depleting water resources and flood events. This research aims to reveal Southern African Development Community disaster risk management strategies for food security to see how they an influence and shape policy at the national level in southern Africa. Sustainable Livelihood approach was adopted as the main theoretical framework for the study. The qualitative Analysis is based largely on data from databases such as national reports, regional reports and empirical findings on the disaster management–sustainable development nexus.

## Introduction and background

Worldwide, the damage from natural disasters has been increasing, with death trends more experienced in developing countries (Kahn 2005). Various scholars agree that natural disaster results in devastating effects in different communities around the globe; they deprive affected persons’ many rights such as the fundamental right to adequate food as stipulated by the 1948 Article 25 of the Universal Declaration of Human Rights (Rukundo et al. 2014; UN 1948). Natural disasters pose a great danger to food security. This is because of a number of factors; such as displacement of people from their settlements, death of people (including breadwinners), decrease in agriculture output and increase in food prices because of price increases. This is detrimental in light of how vital food security is for humans to live a fruitful life.

Food security is one of the major priorities of all countries around the world. Food security is defined as people having ‘access to sufficient, safe and nutritious food which meets their dietary needs and food preferences for an active and healthy life’ (Pirani & Arafat 2016:1). The World Food Summit (1996) defined food security to exist when all people, at all times, have physical, social and economic access to enough, safe and nutritious food that meets their dietary needs and food preferences for an active and healthy life (FAO 2003). In light of the two definitions, Conceição et al. (2016) make conclusive remarks when they note that food security is a core component of the human development and capability paradigm. The scholars note that both food access and entitlement are critical at reinforcing human capabilities.

This research aims to reveal Southern African Development Community (SADC) disaster risk management strategies as a way to influence and shape national policy. This is a research prompted by the recent occurrence of natural disasters in SADC which has had a significant effect on food security. FAO and IFAD (2013) assert that food security indicators revealed that approximately 22.9% of the African population suffered from undernutrition and 28.9% did not have access to adequate supplies from 2010 to 2012 (FAO & IFAD 2013). The world has made some significant progress in addressing basic nutritional needs of the poor and vulnerable groups (United Nations Development Programme [UNDP] 2012:9; Conceição et al. 2016). However, food insecurity remains a threat affecting millions of people, and sub-Saharan Africa accounts for a large share of this problem, with more than one in four people in this region (nearly 218 million) remaining undernourished (UNDP 2012:9).

In recent years, the effects of food insecurity have been felt mostly by countries in southern Africa because of different natural disasters that are affecting the region as illustrated in Table 1.

**TABLE 1:** Food and Agriculture Organisation’s listing of African countries in food crisis requiring external assistance.

Nature of food insecurity Main reasons: Exceptional shortfall in food	Main reasons
Zimbabwe	*Drought-affected 2016 production*: The El Nińo-induced drought resulted in a sharp decrease in the 2016 cereal production and the loss of livestock. An estimated 33% of the rural population require assistance during October–December 2016. This number is projected to increase to 44%, approximately 4 million people, during the peak of the lean period (January–March 2017).
Lesotho	*Drought-reduced 2016 production and higher food prices*: Cereal production declined steeply in 2016, while higher prices are negatively impacting on food access. As a result, almost 709 394 people are food insecure.
Mozambique	*Drought-affected 2016 production and higher food prices*: Drought conditions resulted in lower cereal outputs in southern provinces and in parts of central provinces, while higher prices are adversely impacting food access. Approximately 2 million people are food insecure.
Swaziland	*Reduced cereal output following drought conditions*: The 2016 cereal harvest declined and livestock mortality rates increased on account of the drought conditions. Higher food prices are further affecting the food security conditions, with an estimated 638 251 people in need of assistance.
Malawi	*Sharply reduced cereal production in 2016 and higher food prices*: Maize production in 2016 decreased by one-third compared to the 5-year average, resulting in tighter supplies and higher prices. The number of people requiring assistance is estimated at 6.5 million.

*Source*: Food and Agriculture Organisation (FAO), 2016, *Countries requiring external assistance for food in 36 countries*, p. 1, viewed 01 March 2017, from http://www.fao.org/es/giews/english/hotspots/index.htm

FAO (2016) reveals about five countries in southern Africa that are in need of external assistance for food. There have been continuing droughts and natural hazards which have resulted in these countries being affected. In some cases, this has been attributed to the impact of El Niño-induced droughts that continue to affect agricultural output, the major concern being the reduction in the availability of the cereal production in 2016. In addition, some of the natural hazards that are noted which affect food security included droughts and El Niño, which may have influenced floods. Conceição et al. (2016) note that agriculture also determines food entitlement for the 70% – 80% of poor Africans that rely on its production for income and work. Hence, natural hazards mentioned above pose an imminent threat to food security of most people in southern Africa.

## Impact of climate change on food security

Climate change is at the epitome of natural hazards. Artur and Hilhorst (2014) state that one of the pertinent indicators of climate change is the increase in natural hazards that culminate into disasters. This research can reveal that some of the disasters that are related to climate change include windstorms, floods and droughts and have become more frequent and severe in the southern African region. COP21 United Nations Framework Convention on Climate Change (UNFCCC) meeting in Paris (2015) revealed that many nations are facing food insecurity because of severe climate change hazards. Many scholars have provided a clear evidence of the dangers of climate change (Burke et al. 2010; Georgescu, Lobell & Field 2011; Hertel, Burke & Lobell 2010; Lobell et al. 2011; Mugambiwa & Tirivangasi 2017; Rowhani et al. 2011). Moreover, SADC countries have been affected by water scarcity because of erratic rainfall patterns, increased evaporation and increased water demand in all sectors. Drought-prone areas of Namibia, Botswana and Zimbabwe were found to be more vulnerable to climate change than the more humid areas of Tanzania or Zambia (IPCC 2007).

In 2008, Lobell et al. (2008) noted that southern Africa could lose more than 30% of its main crop; maize, by 2030 because of climate change. It can be noted that climate change affects agriculture which is one of the biggest source for food security in southern Africa. International Labour Organization (ILO) (2009) states that approximately two-thirds of the working class population in Africa still make their living from agriculture. This calls for long-term food security options which need to be taken into account together with the adaptation methods. Climate change affects all three dimensions of food security, namely food availability, food utilisation and food systems stability. Food insecurity occurs when one or more of these three components of security is uncertain or insecure.

## Impact of droughts and floods on food security

Communities in Zimbabwe have been affected by climate change related disasters such as the droughts of 1982, 1991–1992, 1994–1995, 1997–1998, 2002 and 2008 (Bola et al. 2014). In addition, Zimbabwe was also affected by the El-Niño phenomenon, which resulted in the floods of 1997–1998 (Dilley 2000). According to IPCC (2007), floods and droughts are likely to get worse, as it is predicted that the magnitude and frequency of floods and droughts will increase during the 21st century because of changes associated with climate change and variability. This will increase the vulnerability of households that depend on rain-fed agriculture and livestock production for their livelihoods and create new burdens for those communities already poor (Smucker & Wisner 2008).

Recently, the eastern part of SADC has been affected by cyclones, which resulted in extensive flooding, causing economic losses and damage to infrastructure, crops and livelihoods (Munzhedzi 2017). Subsequently, most countries were affected by drought because of changes and the length of the growing season. This led to the decrease in crop yields and low agricultural productivity. The continual year-after-year effects of low rain inputs have led to increased food insecurity within the region. The law of supply and demand states that the lower the supply, the higher the demand for food supplies. This results in less accessibility of food by the poor households as well as climate-related diseases such as malnutrition, cholera and diarrhoea.

## Problem statement

Literature has shown that approximately 90% of southern Africa relies on agriculture in order for them to attain healthy lifestyles (IPCC 2007). Moreover, agriculture accounts for about 70% of employment in the region (IPCC 2007). This high dependence on agriculture makes the region susceptible to weather and climate-related elements such as floods and droughts (Bola et al. 2013). It can be noted that floods and droughts are all linked to excess or insufficient rainfall, river overflow, climate change and human activities (Dilley 2000). In most cases, this is because of both human activities and climate change. Moreover, natural disasters such as floods and droughts can affect crop production, result in loss of life, cause resettlements as well as lack of sustainable development. Moreover, Bola et al. (2013) note that in Zimbabwe alone, floods and droughts resulted in the death of about 800 people, affected approximately 2 million people and displaced about 300 000 people between the year 2000 and 2001.

Although there are many ways in which communities have adapted to the effects of natural hazards. This article seeks to analyse SADC disaster risk management strategies as a way to influence and shape policy at national level within the region. Research has revealed that climate change impacts on agriculture and climate policies are either global or regional. However, the impacts and costs of climate change cannot be optimally determined on a global basis as the climatic impacts differ from region to region, between countries or even within a country. Thus, this researcher has chosen to assess problem of natural disaster at regional level. The understanding of SADC risk management mechanisms in place will help to identify ways in which such instruments can be used to effect national policies. This research will look at the SADC disaster risk management frameworks which may or can influence national policies of its member states on how to deal with climate change and its related disastrous effects on food security.

## Objectives of the study

The overall objective of this article is to solve the problem of food insecurity in southern Africa in light of natural disasters which are on the increase within the region. Moreover, meeting the objectives will result in sustainable development in Africa as well as in many parts of the world.

## Theoretical framework of the study

This study adopts the Sustainable Livelihoods Approach (SLA) in order to understand how people may cope with natural disasters in southern Africa. The researcher can reveal that SLA has often been adopted for disaster risk reduction as evidenced by the following studies: Dibben and Chester (1999), Glaser (1996), Kelman and Mather (2008), Tobin and Whiteford (2006). These studies were done in the European context, and hence, because of the rise of natural disasters, the researcher decided to adopt SLA. Chambers and Conway (1992) propose:

A livelihood comprises the capabilities, assets (stores, resources, claims and access) and activities required for a means of living: a livelihood is sustainable which can cope with and recover from stress and shocks, maintain or enhance its capabilities and assets, and provide sustainable livelihood opportunities for the next generation; and which contributes net benefits to other livelihoods at the local and global levels and in the short and long term. (p. 5)

More similarly, the researcher adopts the application of SLA to natural disasters as stipulated by Kelman and Mather (2008). The two scholars used this theory in the study of volcanoes, but given the strength and effectiveness of the theory, the author finds it useful to apply the same approach to the management of natural disasters in southern Africa. Kelman and Mather (2008) note that SLA can be applied in four ways:

(1) understanding, communicating and managing vulnerability and risk and local perceptions of vulnerability and risk beyond immediate threats to life

(2) maximising the benefits to communities of their disaster environment, especially during quiescent periods, without increasing vulnerability

(3) managing crises

(4) managing reconstruction and resettlement after a crisis. (p. 193)

### Managing vulnerability and risk

The first application of SLA to natural disasters is understanding, communicating and managing vulnerability and risk along with local perceptions of vulnerability and risk beyond immediate threats to life. This includes looking at the immediate dangers of the disaster to human life as well as property, infrastructure, livestock, crops and the immediate threat to food security. This is what should be in the mind of planners as they encounter disasters.

### Maximising community benefits sustainably

The second application of SLA to natural disasters is maximising the benefits to communities within the environment during this period, while decreasing vulnerability. This is a period of assessment where policy-makers can work on understanding how vulnerable communities are in such situations. For instance, in the case of Mozambique, disagreements ensued between the government of Mozambique and international donors, notably UN-Habitat and Department for International Development (DFID). The international donors promoted a ‘Living with the Floods’ strategy which suggested flood management practices that encouraged people to live on flood prone areas and take advantage of the fertile soils that result from the floods.

The agencies argued that there was a need to develop Early Warning Systems (EWS) which would allow people to vacate their households once a flood was noted. The international donors noted that people in Mozambique did not have an alternative because most of them were living in poverty. Thus, the only choice available was to live with floods. On the contrary, Artur and Hilhorst (2014) note that the government of Mozambique, however, favoured a ‘flood free’ approach which stresses the need to resettle people living on the floodplains. The case study reveals the need to look at all alternatives when it comes to disaster risk management.

### Managing crises

The third application of SLA to volcanoes is managing crises. This is where countries affected have to look at the measures in place which deal with emergency situations. In most cases, unpreparedness, lack of funds and support from the international community and the national government may result in grave consequences. Consequently, there is a need to look at stakeholders who can help, prevent natural disasters from happening.

### Managing reconstruction and resettlement

The fourth application of SLA to natural disasters is managing reconstruction or resettlement after a volcanic crisis. There are two alternatives: (1) adopting an all-hazards and all-vulnerabilities approach and (2) opting for resettlement. An all-hazards and all-vulnerabilities approach results in the victims living near the affected area. However, some would move to adopt a resettlement measure where people are removed from affected areas to environments which are not prone to hazards.

## Methodology

The researcher adopted an extensive literature review from peer-reviewed literature, conference papers and reports from SADC documents related to climate change and climate change related disasters, food security and malnutrition. A snowball searching technique was informed by the criteria indicated by Wohlin (2014), and the researcher did the following:

- Search terms were executed in Google Scholar and the publications that came up were analysed.
- The relevant and highly cited (as indicated by the number of citations) publications were then searched by title and author.
- The authors who were cited by the selected authors were searched for and new authors who came up were continually searched in the Web of Knowledge and Google Scholar.

This sampling procedure ensured that the most relevant information was made available to the researcher as revealed in the presentation of results and discussion. Through an extensive literature analysis from previously published articles, the study seeks to gain an understanding of the impacts of natural disasters on food security and the researcher went on to look at the role of SADC in dealing with such climate change related disasters. Data were analysed qualitatively using content analysis where emerging themes and sub-themes were presented in a consecutive order.

## Results and discussion

The SADC region, like other regional blocks, deliberates on the most pressing issues facing the region. They reckon that disaster risk management strategies should include the following: preparedness, mitigation, response, rehabilitation and recovery. This research looked at the SADC disaster risk management frameworks, which may or can influence national policies of its member states on how to deal with climate change and its related disasters on food security.

## Monitoring and climate predicting services

The region established a regional climate services centre, which provides regional services for monitoring and predicting extreme change in climatic weather conditions. The role of the centre is to develop and disseminate information on meteorological, environmental and hydro-meteorological products (SADC 2011). The centre works with universities, research scientists, national, regional and international climate centres worldwide. The centre has trained personnel who work for National Meteorological and Hydrological Services (NMHSs). The regional climate services centre has created climate predicting methods that cover the end-users in the various weather-sensitive economic sectors such as agriculture, health, energy, water resources management and transport in the region. This department was established in 1990 and it is headquartered in Gaborone, Botswana. SADC attracted funding from UNDP, World Meteorological Organisation (WMO), World Bank, agencies of the United States of America such as National Oceanic and Atmosphere Administration-Office of Global Programmes (NOAA-OGP) and United States Agency for International Development (USAID), the Kingdom of Belgium and others (SADC 2011).

Per SLA, one of the most essential strategies of managing disaster is to manage vulnerability and risk. This centre has a vital role to play in ensuring that food security is attained within the region. It is the predictive means and use of various technological services that can help different states in avoiding risk and vulnerability. This centre has an ability to influence how policies are framed in individual countries. However, its effectiveness can only be ensured through sufficient funding, more human capital and an increase in the use of its services. In addition, risk in individual countries can be reduced by transferring information within the participating countries on issues of climatic change and its impact; providing advice and guidance to the participating countries on the establishment and strengthening of monitoring and forecasting capabilities for droughts and floods at national and regional level; and producing specialised maps to show the magnitude, extent and probability of occurrence of hazardous weather phenomena such as drought, floods and tropical cyclones; and the centre can as well help in forging a way forward on how to adapt to different climatic hazards (SADC 2011). This can be ensured by playing an advisory role to different countries within the region, who have a chance or higher probability of being affected by different natural disasters.

World Meteorological Organisation, Global Facility for Disaster Reduction and Recovery (GFDRR) and Shanghai Meteorological Service (SMS) 2016 workshop identified the impact-based forecast and warning services as a priority for WMO members to increase the relevance and utility of their NMHSs’ forecasts and warnings. The impact-forecasts emphasis reveals what the impact of the hazard will be, thus increasing the preparedness of different countries within the SADC region. Moreover, using the lenses of SLA, one can note that SADC regional climate services centre can play an advanced role in performing risk-based warnings, which may include the following: time of day, time of year, the hazard and the probable impacts of the hazard on rural and urban spaces. Consequently, providing these kinds of services will results in the preparedness of different countries within the region. Moreover, the activities of this centre will increase the adaptation and the ability of individual countries within SADC to mitigate the effects of climate change related disasters such as floods and droughts. Therefore, food security will be ensured through the ability of different countries to prepare themselves for any form of disaster.

## Regional Vulnerability Assessment and Analysis programme

The Regional Vulnerability Assessment and Analysis (RVAA) programme is one of the instruments used by SADC in coping with various disasters. This regional block continues to reckon that livelihood vulnerability and food insecurity are prominent features of the poverty that afflict much of its population. With the existence of climate change hazards such as floods, food security continues to be threatened. Hence, the adoption of Vulnerability Assessment and Analysis (VAA) programme (SADC n.d.). This was engineered in a bid to create strategies which can reduce the vulnerability of different countries within the region. In 1999, SADC formed the Regional Vulnerability Assessment Committee (RVAC) with the intention of monitoring of states’, households’ and individuals’ capacity to deal with external hazards such as drought, economic crises and climate change (SADC n.d.). Further, VAA initiative is meant to increase the coping strategies considering the impending or active disasters within SADC.

The VAA initiative is implemented both at regional and national levels. At country level, they are termed National Vulnerability Assessment Committees (NVACs). They are multi-sectorial committees which involve government ministries, non-governmental organisations (NGOs) and international organisations involved in poverty reduction and socio-economic development. The major role of NVACs is to carry out vulnerability assessments, both annually and periodically. Moreover, they conduct special studies on thematic areas such as nutrition, climate change, food safety and security (SADC n.d.). The RVAA has been more specifically designed to track, report and respond to food insecurity in the region. This is what makes the programme to be very important to this research aimed at ensuring food security in the age of climate change disasters.

The researcher used SLA lenses to understand how RVAA programme helps different countries to deal with natural disasters and ensure food security. SLA advocates that countries ought to be able to understand, communicate and manage the vulnerability of their immediate population. Vulnerability is defined as the susceptibility of exposed elements such as people, their livelihoods and property, to suffer adversely when affected by a hazard. Hence, through the effective use of RVAA programme and NVACs, the impacts of climatic hazards may be reduced. This is attained through the preparedness of SADC member states, which can be ensured by reliance of scientific research carried out by both NVACs and RVAA within the region and individual countries, respectively.

Moreover, maximising the benefits to communities of their disaster environment without increasing vulnerability is another aspect of SLA, which is essential to revealing the importance of RVAA programme to SADC countries. In every hazard that occurs, there are benefits that may be gained from that area. For instance, in the case of Mozambique, donors were advocating for the ‘Living with the Floods’ strategy, which suggested flood management practices that encouraged people to live on flood prone areas and take advantage of the fertile soils that result from floods. In contrast, the Mozambican government advocated for flood-free strategy. This is where RVAA and NVACs experts’ opinion would have proven vital. This can be achieved after understanding the food security needs of their population and how vulnerable the population is in the wake of the disaster.

## Southern African Development Community response to climate change

Southern African Development Community, like all the other regional groups around the world, has a history in tackling the effects of climate change. This was largely influenced by the development of policies relating to climate change by the United Nations. It can be traced back to the drafting of UNFCCC and the Kyoto Protocol. These are the two documents that led the foundation on importance of developing climate change mitigation and adaptation strategies. SADC as a regional bloc reckons that there is a need to develop policies to tackle challenges. Barnard (2014) states that:

Article 5(2)(a) of the *Treaty of the Southern African Development Community*, 1992 (SADC Treaty) states that for the region to attain the objective of sustainable development, the harmonisation of political and socio-economic policies is necessary. (p. 29)

Hence, this article reckons that the development of climate change mitigation strategies has a huge impact on ensuring food security within the SADC region. In 2003, SADC developed the *Regional Indicative Strategic Development Plan* (*RISDP*). This plan acknowledges climate change as an environmental hazard. Furthermore, in 2010, SADC introduced the Southern Africa Climate Change Framework. This framework proposed a set of adaptation programmes, for instance, disaster risk reduction and management, sectorial planning and implementation and building economic and social resilience (SADC-CNGO Policy Paper 7, 2012). Although Bernard (2013) realised that frameworks can only guide different states on the way forward, they are not legally binding. However, they serve as recommendations to SADC countries on how to respond to climate change in the region. Furthermore, a framework serves to reveal the priority of the SADC countries. Consequently, SADC developed different frameworks, strategies or initiatives to ensure that member states respond to natural disasters. The controlling of natural disasters has a significant effect in promoting food security in the region.

## Southern African Development Community climate change adaptation strategy

In 2008, SADC developed climate change adaptation strategy (CCAS) during a Multi-Stakeholder Dialogue in Lesotho. The main aim of the strategy was to improve climate resilience in southern Africa through improved integrated and adapted water management at regional, river basin and local levels (DEA 2014). The water management strategy was to ensure that the region could cope with climate change variability experienced within the SADC region. It should be noted that SADC managed to create Regional Climate Change Programme (RCCP), which ran from 2009 to 2014 and was aimed at conducting water resources assessment of climate change impacts on trans-boundary basins (DEA 2014; SADC 2011). However, this article discusses CCAS because it was designed to guide the SADC region for the next 20 years. This means that, at the present moment, the resolutions which were adopted in Lesotho still matter. It pioneered the adaptation strategies being undertaken within the SADC region at present. SADC CCAS is a multi-dimensional approach, which ensures that adaptation takes place at different levels. Therefore, measures occur at different levels, areas of adaptation or prevention. Hence, it gives the countries in SADC the autonomy to create relevant adaptation strategies necessary for each environment. SADC CCAS is an important approach in the battle against food insecurity in the region. It ensures timely intervention because of the adoption of climate change resilient strategies adopted in different countries.

The objective is to promote further the application of integrated water resources management as a priority tool to reduce climate vulnerability and to ensure that water management systems are well adapted to cope with increased climate variability. The adaptation measures which were adopted are based on three levels of intervention, namely, governance, development and management. Firstly, *water governance*, which includes the following: making an average citizen to become aware of climate change and its impact, extensive research on climate change and development in order to create local solutions and creating national policies on water management (DEA 2014; SADC 2011). The second area of intervention is *infrastructure development*, which consists of the following: increased water storage, thorough construction of reservoirs and protection of water sources; water supply and sanitation; improvements of drainage and irrigation systems to ensure food security; and reduction of the effects of floods within the region (DEA 2014; SADC 2011). Lastly, intervention was done through water management. Focus was directed on specific areas such as developing systems to share data and store information which can influence decision-making and policy; incorporating climate uncertainties into planning, policy-making and decision-making; determining groups, places, and ecosystems which may be affected by the effects of climate change; and developing the EWS which can be used to predict and forecast the occurrence of climate change related disasters; and members of SADC should be able to develop integrated water resources, water demand and quality management (DEA 2014). The use of EWS by individual countries within SADC will ensure that countries are ready for any impending drought or rain shortages in the future. In this case, it is pertinent for all countries within SADC to implement EWS as a national policy.

## Conclusion

This research reveals that most of the climate change hazards have a serious implication on food security. The only way to ensure food security at country levels is achieved through dealing with climate-related disasters and hence the need to adopt the strategies discussed in this article. SADC as a region has developed strategic adaptation and mitigation mechanism which provide an insight on how its members should progress into the future. There is ample evidence of research into the development of the way forward; what is left is the implementation of these policies. Further, all the strategies discussed in this article were justified by SLA, which the researcher believes is vital for all countries that are facing climate-related natural disasters. It is the careful management of disasters through adaptation, which would ensure that food security is ensured in SADC.
